# Chronic Progressive Spastic Paraparesis: Think of Peroxisomal Disorders - A Case Report of X-Linked Adult Onset Adrenoleukodystrophy With an Update on The Latest Treatment Strategies

**DOI:** 10.7759/cureus.9626

**Published:** 2020-08-09

**Authors:** Hassan Kesserwani

**Affiliations:** 1 Neurology, Flowers Medical Group, Dothan, USA

**Keywords:** spastic paresis, allogenic bone marrow transplant

## Abstract

The etiology of chronic progressive myelopathy can be a challenging diagnostic dilemma, especially in the absence of a structural lesion of the neural axis or a conspicuous inflammatory process. The differential diagnosis can be pleomorphic. However, the emergence of signs of adrenal dysfunction, lack of a structural lesion of the spinal cord and the emergence of cerebral demyelination should raise the suspicion of X-linked adrenoleukodystrophy (ALD). The biochemical signature of elevated serum very long chain fatty acids and a pathogenic mutation of the ATP-binding cassette subfamily D1 (ABCD1) gene is pathognomic. We present an adult variant case that marched through the classic catena of clinical syndromes: subtle adrenal dysfunction, chronic progressive myelopathy and ultimately cerebral demyelination. We outline the biochemistry, clinical semiology, pathology and therapeutic challenges in this group of patients. The unfolding disease in children and young adults can be arrested with allogenic and more recently autologous hematopoetic bone marrow transplantation. The challenge of therapy in adult patients with chronic progressive myelopathy who develop cerebral demyelination remains a therapeutic dilemma. Our case highlights the nuances of managing this group of patients and alerts the clinician to early diagnosis prior to the emergence of cerebral demyelination.

## Introduction

X-linked adrenoleukodystrophy (ALD) is a peroxisomal disorder, an inborn error of metabolism that is monogenic. Despite displaying six phenotypic presentations, all patients carry mutations in the adenosine triphosphate (ATP)-binding cassette subfamily D1 (ABCD1) gene and accumulate very long chain fatty acids (VLCFAs) in the plasma and in all tissues [1]. Plasma concentrations of C26:0 and C26:C22 and C24:C22 ratios are increased [2]. The fundamental biochemical lesion is impaired peroxisomal beta-oxidation of VLCFA. Impaired processing of VLCFA leads to accumulation of VLCFA-coenzyme A esters in the cytosol. The elevated levels of VLCFA esters lead to toxicity of adrenocortical cells, glial cells (astrocytes and oligodendrocytes) and neurons [3]. Exposure of adrenocortical cells to VLCFA esters leads to reduced stimulation by adrenocorticotropic hormone (ACTH) [4]. Exposure of neural cells to VLCFA esters leads to cytosolic calcium accumulation, mitochondrial depolarization and subsequent neurotoxicity [5]. Injection of C24:0 complexed to phospholipids into the cerebral cortex of mice leads to microglial activation and apoptosis of neurons [6].

Pathologically, the topography of findings depends on the phenotype. Variably, there is generalized cerebral atrophy and demyelination of the splenium of the corpus callosum and the parieto-occipital white matter. In the spinal cord, there is loss of myelin and axons in the corticospinal tracts, gracile fasciculus and spinocerebellar tracts. Microscopically, lesions display three layers of pathology; in the outermost layer, there are lipid laden macrophages with demyelination and axonal sparing, in the middle layer; there are macrophages, myelinated fibers, demyelinated fibers and perivascular mononuclear cells; and the innermost layer displays a dense mesh of glial fibrils [7]. At the microstructural level, there are cytoplasmic inclusions that differ between brain macrophages and parenchymal cells (adrenocortical cells and testicular Leydig cells). These are pathognomic linear cytoplasmic inclusions that exist in trilaminar sheet-like structures. In brain macrophages, they exist in discrete membrane bound structures. In the parenchymal cells, they exist free in the cytosol with cleft-like spaces [8]. These inclusions are made of VLCFA esters.

Virtually all males develop adrenocortical deficiency early on in the disease and evolve into a chronic progressive myelopathy. Once there is cerebral involvement, the disease is inexorably progressive. In women, the disease develops later in life, in their 60s, and rarely involves the adrenal glands or brain. The core phenotype is adrenal insufficiency with chronic progressive myelopathy. Approximately 40% of young adult patients below 18 years of age develop progressive cerebral demyelination and 10% of adult males do [9]. 

We present a patient with classic adult onset progressive spastic paraparesis who progressed to a wheelchair, with bladder atony requiring suprapubic catheterization, adrenal dysfunction and cerebral demyelination with cognitive impairment. He had the fundamental dual pathognomic abnormalities, pathogenic ABCD1 mutation and elevated serum VLCFA. The patient was referred for evaluation of potential allogenic bone marrow transplantation. We outline the case in detail and in the discussion we flesh out the clinical semiology of X-linked ALD and outline the evolving treatment strategies.

## Case presentation

We report the case of a 62-year-old highly intelligent retired pilot, who at the age of 37 years noted bladder urgency when he stepped off his aircraft. While he was serving in Bosnia, he developed low-grade back pain and numbness in the feet. By the age of 40 years, he was dragging his feet. He then noted oscillations of his right ankle, clonus and jerking of his legs. He was diagnosed with cervical spondylotic myelopathy and underwent a cervical cord decompression. He did not improve; his feet and toes continued to feel numb and he continued dragging his feet. By the age of 49 years, he started using a cane. His legs continued to feel stiff. Meanwhile, he never complained of hand numbness or upper extremity symptoms until recently. He continued to worsen until the age of 56 years, when he developed bladder retention and he started using a scooter at his daughter's wedding, especially for long distance ambulation. By the age of 60 years, a suprapubic catheter was installed. One year later, he was bound to a wheelchair. Over the last two years, his cognition started deteriorating, with forgetfulness, searching for words, difficulty balancing his check book and problems troubleshooting with his computer skills. His reading fluency diminished with respect to processing information, and he was forced to read a sentence multiple times before he grasped its meaning. He also entered into a bowel program with Dulcolax water-based suppositories. Over the last five years, he has also developed fatigue and lack of stamina and blood pressure volatility; specifically systolic blood pressure varying from 90 to 150 mmHg. He also developed thinning of his scalp hair and loss of lower extremity hair. More recently, he has developed a very mild dysarthria with a deliberate attempt at slowly imbibing water for fear of choking. Over the last year or two, he has noted problems with manual dexterity, especially with his right hand, such as fluidity of penmanship and grip power.

His past medical history was significant for a pulmonary embolus and atrial fibrillation. There was no definitive family history; his maternal grandfather had reduced mobility and choking with food later in his life. However, the details are limited. His medications included ropirinole 2 mg twice a day and valium 2.5 mg at night, on as needed basis, for the lower extremity spasm and stiffness. He was also on amiodarone and xarelto for atrial fibrillation.

On examination, his blood pressure was 136/69 mmHg and pulse of 94 beats/minute. On inspection of his scalp, hair thinning was obvious but no skin bronzing was noted. He was in a wheelchair, which he was able to propel with his arms. He required help to stand and transfer. He was mentally sound and answered questions with ease. His delayed memory was 3 out of 3 at five minutes. His digit span was 7 out of 7. Visuomotor skills were preserved with simple pantomime. No evidence of limb-kinetic apraxia or ideomotor apraxia was noted with coin deftness or transitive acts, such as unlocking a door with a key, respectively. His speech was fluent with understanding and repetition intact grossly. Cranial nerve evaluation revealed full ocular motion without ptosis and with intact accommodation. No facial weakness or tongue weakness was noted. Hypophonia and dysarthria were absent, and shoulder shrug was perfectly normal.

Motor examination with Medical Research Council (MRC) grading was 5/5 for both upper extremity muscles, including deltoids, biceps, triceps, brachioradialis, brachialis, pronators, supinators, wrist extensors and flexors,except finger spreaders that were graded at 3/5 with the right hand and 4/5 with the left hand. Power grip was noticeably diminished with the right hand. The lower extremities power was graded at hip flexors 2/5 symmetric, knee extensors 3/5 symmetric, hamstrings 3/5 symmetric, ankle dorsiflexors and plantar flexors 2/5 symmetric and toe extensors 2/5 symmetric. Upper extremity reflexes were graded at 2, but knee deep tendon reflexes (DTRs) were graded at 4+ and ankle DTRs at 4+ with bilateral ankle clonus. He displayed bilateral lower extremity spasticity and Babinski sign bilaterally. 

**Figure 1:**
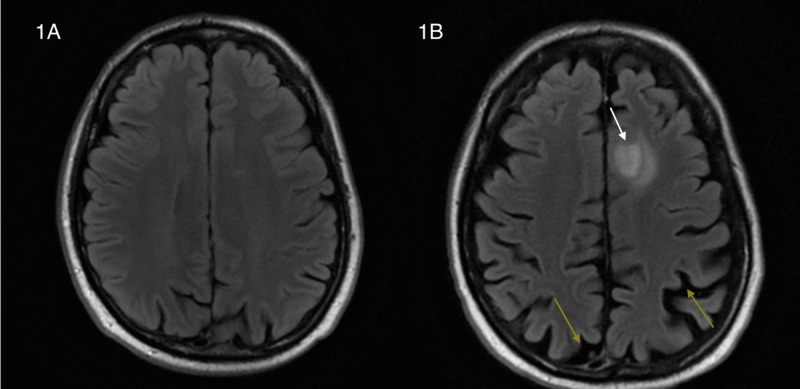
Axial FLAIR MRI of the brain. (A) Normal axial FLAIR MRI image two years prior. (B) Axial FLAIR MRI image demonstrating mostly bilateral parieto-occipital atrophy (yellow arrows) and large left frontal lobe plaque (white arrow). FLAIR, fluid-attenuated inversion recovery

An MRI of the brain and whole neural axis revealed predominantly bilateral parieto-occipital brain atrophy with a large right frontal lobe plaque. We were also able to document the evolution of cerebral atrophy and demyelination over the course of two years (Figure 1).

**Figure 2:**
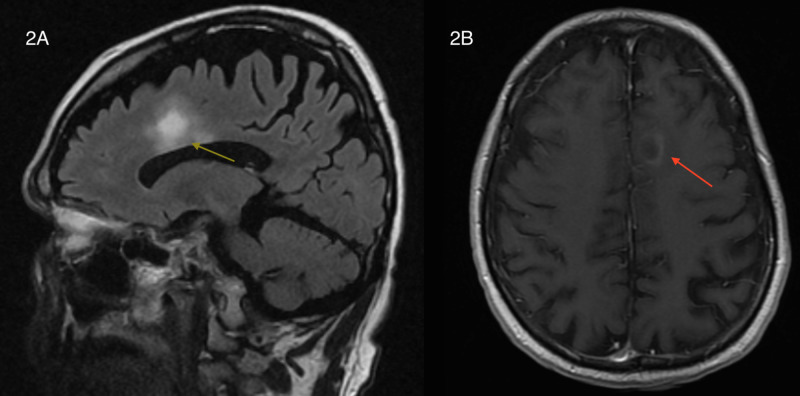
(A) Sagittal FLAIR MRI image demonstrates extension of the large right frontal lobe plaque into the corpus callosum (yellow arrow). (B) T1-weighted axial MRI of the brain with gadolinium enhancement shows peripheral ring enhancement of the right frontal lobe lesion (red arrow). FLAIR, fluid-attenuated inversion recovery

Sagittal fluid-attenuated inversion recovery (FLAIR) MRI images reveal extension of the right frontal lobe lesion into the anterior corpus callosum and T1 axial MRI images with gadolinium enhancement shows ring enhancement of the right frontal lobe lesion (Figure 2).

Cerebrospinal fluid analysis was normal for protein, white cell count differential, glucose, oligoclonal bands and IgG index. Serum human T-cell lymphotropic virus (HTLV-1) type I antibodies were negative. A hereditary spastic paraplegia panel for 42 genes was also negative. Serum VLCFA reveals elevated C26:0 and elevated ratios of C24/C22 and C26/C22, the biochemical signature of X-linked ALD (Table 1).

**Table 1:** Table demonstrating elevated serum C26:0 very long chain fatty acids and elevated serum C24/C22 and C26/C22 ratios, the biochemical signature of X-linked adrenoleukodystrophy.

Serum marker	Level	Normal range
C26:0	3.3 (high)	0.31-0.81 mmol/liter
C24/C22 ratio	1.67 (high)	0.726-0.988
C26/C22 ratio	0.07 (high)	0.0049-0.0118

An ACTH stimulation test was negative for adrenocortical insufficiency, but this may be false negative and a second test is pending. A whole exome sequencing (WES) test was carried out and a pathogenic variant was detected in the ABCD1 gene as an in-frame duplication of seven amino acids in the BSCL2 gene, a unique mutation. This was not detected in a large population of cohorts [10]. This variant was observed in a hemizygous state. The clinical presentation of a chronic myelopathy, with recent cerebral demyelination, elevated VLCFAs and a pathogenic mutation in the ABCD1 gene, is diagnostic of X-linked ALD. With these data, the patient was referred for possible qualification into an allogenic bone marrow transplant program.

## Discussion

The clinical presentation of X-linked ALD is variable, can start in childhood or begin in adulthood. Adrenal dysfunction is inconstant, but ultimately many develop a progressive myelopathy with bladder urgency and variable peripheral neuropathy and cerebral dysfunction (Table 2). There are six presentations that are not mutually exclusive: Addison-only, childhood, adolescent and adult, adrenomyeloneuropathy with or without cerebral demyelination, and asymptomatic or symptomatic carriers [10]. 

**Table 2:** Spectrum of clinical presentation of X-linked adrenoleukodystrophy (ALD) with relevant markers: adrenocorticotropic hormone (ACTH) and attention deficit hyperactivity disorder (ADHD).

Adrenal dysfunction	Urogenital	Myelopathy	Cerebral	Other
Fatigue	Bladder urgency	Spastic gait/spastic legs - may progress to wheelchair	Variable cognitive dysfunction	Mild neuropathic symptoms
Hypotension	Testicular dysfunction/hypogonadiam	Cramps	Mental slowing	Asymptomatic carriers
Bronzing of skin		Eventually atonic bladder requiring suprapubic catheter	ADHD/behavioral issues/impaired vision and hearing in childhood	Females may present after age 60 years/with myelopathy/rarely adrenal/very rarely cerebral
Thinning of hair/balding			Psychosis/frontal lobe behavior in adults	
Impaired ACTH stimulation test			Focality; visual agnosia, hemiparesis, dysarthria and dysphagia with advancing disease	
			Demyelination typically in splenium of corpus callosum and occipital lobes	

As outlined above, the evolution of symptoms is from adrenal insufficiency to chronic progressive myelopathy and eventually to cerebral demyelination, especially in males before the age of 60 years. Once cerebral demyelination sets in, the disease is irreversibly progressive. The idea behind current therapy is to prevent the development of cerebral demyelination. Allogenic (donor) hematopoietic stem-cell therapy (allo-HSCT) was the mainstay of therapy for boys with adrenal insufficiency or mild symptoms to prevent the development of cerebral demyelination [11,12]. Gene therapy with autologous hematopoietic stem cells therapy (auto-HSCT) has been investigated as an alternative to allo-HSCT (Table 3). The latter is limited by graft failure and graft-versus-host-disease. Auto-HSCT involves infusing autologous CD34+ hematopoietic stem cells (obtained by apheresis), transduced ex vivo with a lentiviral vector that contains ABCD1 complementary DNA (cDNA). Patients are first conditioned with busulfan and cyclophosphamide, after which the transduced CD34+ cells are infused. A total of 17 boys received auto-HSCT; 88% were alive and with minimal functional disability with minimal clinical symptoms at a median follow-up of 29.4 months (range, 21.6 to 42.0) [13].

**Table 3:** Comparison of allogenic hematopoetic stem cell transplant (allo-HSCT) and autologous hematopoeitic stem cell transplant (auto-HSCT). GVHD, graft versus host disease; N/A, not applicable

Allo-HSCT	Auto-HSCT
Requires immunosuppression	Low risk of mutagenesis due to viral introduction
Graft failure 6%-14%	N/A
GVHD 8%-45%	N/A
Progression common on MRI brain in year 1	Low risk of progression on MRI brain in first year

Both treatment modalities are highly effective with disease stability at two years, but more patients with allogenic transplant had brain MRI progression in the first year [11,12]. In adults, auto-HSCT is limited by body weight as amount of virus production for ex vivo gene therapy is a limiting factor. Hence, our patient was referred by allo-HSCT.

## Conclusions

We present a case of chronic progressive myelopathy with subtle background adrenal dysfunction, who eventually progressed to the cerebral demyelination phase of the neurometabolic disease, X-linked ALD. This case report highlights the need to check for serum VLCFAs in patients with unexplained progressive myelopathy. Furthermore, the onus is on the clinician to diagnose this disease early on before the onset of cerebral demyelination, as the disease can be preemptively arrested with allo-HSCT or auto-HSCT. Being of monogenic cause, in the future this disease is potentially amenable to other forms of therapy such as gene silencing, anti-sense oligonucleotide therapy, and gene editing, such as clustered regularly interspaced short palindromic repeat (CRISPR) technology. Furthermore, we highlight the dual diagnostic signature of elevated serum VLCFA and a genetic mutation of the ABCD1 cassette gene in X-linked ALD. In our case, we demonstrate a hitherto undefined mutation, which was not present in a cohort of a normal population, a frequent diagnostic pattern in the WES diagnostic technology.
